# Vale Neil McKern – Australian Insulin Receptor Scientist

**DOI:** 10.3389/fendo.2014.00105

**Published:** 2014-07-03

**Authors:** Colin Wesley Ward, Michael Colin Lawrence

**Affiliations:** ^1^Walter and Eliza Hall Institute of Medical Research, Parkville, VIC, Australia; ^2^Department of Medical Biology, University of Melbourne, Parkville, VIC, Australia

**Keywords:** Neil McKern, insulin receptor, type 1 insulin-like growth factor receptor, epidermal growth factor receptor, ErbB2

Neil Moreton McKern, the first author on the landmark paper describing the crystal structure of the human insulin receptor ectodomain (1), passed away on the 20 March 2014 after a year-long battle with multiple myeloma.

Neil McKern was born on 17 December 1946 at Richmond, New South Wales. His career in science began at the age of 18 as a technical assistant to Dr. Fred Stewart in the CSIRO Division of Protein Chemistry in Parkville, VIC, Australia, where he worked on the chemical synthesis of the peptide hormone oxytocin. Neil then studied part time, obtaining a B.Sc. degree in 1971 and a Ph.D. degree in 1981, both from the University of Melbourne. His thesis title was “*Serum somatomedin and the regulation of growth-studies with rodent models*.”

Over the subsequent 10 years at CSIRO, Neil contributed to several aspects of protein science, including the purification of wool proteins for structure determination, the sequencing of the pilin protein of *Dichelobacter nodosus* (which causes footrot in sheep), the study of the proteins of infectious bursal disease virus (a major poultry pathogen) coupled with development of a recombinant vaccine, and the study of variation in the coat protein sequence across the *Potyviridae* (the largest family of plant viruses). These projects reflected the agricultural priorities of CSIRO’s science at the time.

**Figure 1:**
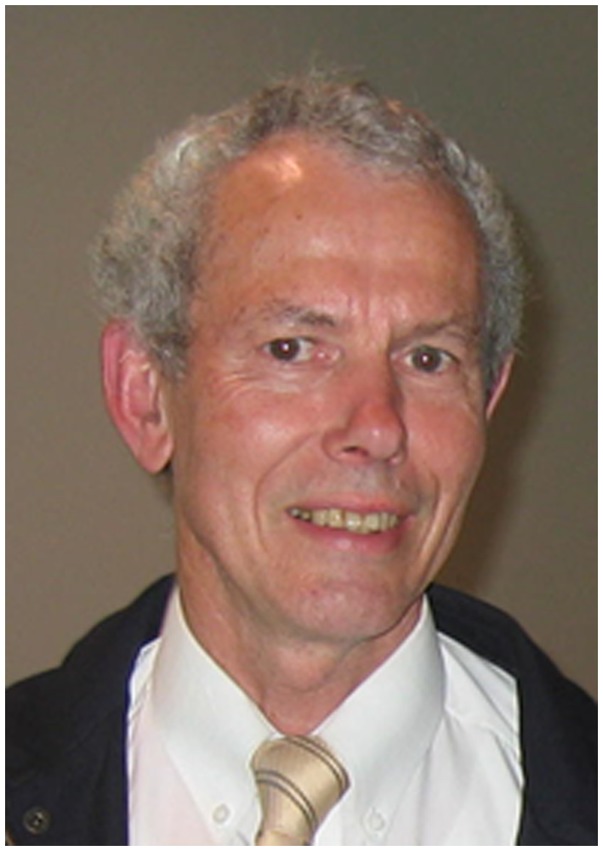
**Neil McKern in 2006**.

These endeavors laid the foundation for Neil’s involvement starting in 1992 with what was to become a “grand challenge” at CSIRO, namely, Colin Ward’s program to understand the structural biology of the insulin receptor, the Type 1 insulin-like growth factor, and the epidermal growth factor receptor. Spurred on by the successful determination of the structure of the human growth hormone bound to its receptor by Tony Kosiakoff and colleagues at Genentech, the allure of a structure of insulin bound to its receptor was irresistible. However, the complexity of these receptor ectodomains proved to be substantial – high levels of glycosylation, high numbers of disulfide bonds, and the existence of multiple liganded states. Neil masterminded the practicalities of purifying bulk quantities of the ectodomains and truncated ectodomain fragments of these receptors from large (100 l plus) scale mammalian cell culture supernatants. His demands were high – to achieve tens of milligram quantities of receptor protein displaying no more than one band on an isoelectric focusing gel. He set up the first forays of the lab into robotic protein crystallization and the combination of this technology with isoelectrically pure protein finally delivered the first – and still only – structures of the Type 1 IGF-1 receptor L1-CR-L2 fragment (2), the insulin receptor L1-CR-L2 fragment (3), and the intact insulin receptor ectodomain (1). Such care and ingenuity absorbed Neil until 2007, at which point CSIRO terminated the project upon Colin’s retirement. His endeavors also led to structures of the truncated ectodomains of the epidermal growth factor receptor in complex with TGFα (4) and of its co-family member ErbB2 (5). Neil retired from CSIRO in 2010, but not before playing a key role in ensuring that the structural studies of the insulin receptor continued at the neighboring Walter and Eliza Hall Institute of Medical Research in Melbourne.

Always striking to all Neil’s colleagues was his cheerful optimism, which was coupled with healthy cynicism about the way in which his employing organization functioned. Part of this optimism derived no doubt from his passion for running, which seemed to not only energize his body but also to provide a wealth of ideas about how to prepare the next sample for crystallization. Giving up on the “grand challenge” would have to Neil been akin to pulling out of a marathon half way along the course.

Neil was dedicated to his family, which together faced the challenge of re-building their beloved family retreat in Marysville after it burnt to the ground in the devastating Black Saturday bushfires of 2009.

For us, and for the many others who worked with him, his enthusiasm for science, and his collegial support is greatly missed.

## Conflict of Interest Statement

The authors declare that the research was conducted in the absence of any commercial or financial relationships that could be construed as a potential conflict of interest.
